# Supplementary material for: Practical Models of Pharmaceutical Care for Improving Tuberculosis Patient Detection and Treatment Outcomes: A Systematic Scoping Review
Source: Trop Med Infect Dis. 2023 May 20;8(5):287. doi: 10.3390/tropicalmed8050287 (PMC10224363; doi:10.3390/tropicalmed8050287)
Supplement: Supplementary file 1 [file tropicalmed-08-00287-s001.zip › tropicalmed-2258493-supplementary.pdf]

Supplementary Material

Supplementary File S1. The key terms for the search strategy in the Pubmed and Cochrane databases

Pubmed:

((((((("Pharmacy"[Mesh]) OR "Pharmacies"[Mesh]) OR "Pharmacy Service, Hospital"[Mesh]) OR "Pharmacy Technicians"[Mesh]) OR "Community Pharmacy Services"[Mesh]) OR ("Pharmacy Research"[Mesh] OR "Pharmaceutical Services, Online"[Mesh] OR "Pharmaceutical Services"[Mesh])) AND (("Tuberculosis"[Mesh]) OR ("Latent Tuberculosis"[Mesh] OR "Tuberculosis, Multidrug-Resistant"[Mesh])).

Cochrane Library:

**Search Code**	**Key terms**	**Total article identified**
#1	[Pharmacies] in all MeSH products	131
#2	[Pharmacy Service, Hospital] explode all trees	158
#3	[Pharmacists] explode all trees	727
#4	[Pharmacy Technicians] explode all trees	21
#5	[Pharmaceutical Services] explode all trees	1931
#6	[Tuberculosis] explode all trees	2621
#7	[Latent Tuberculosis] explode all trees	141
#8	#1 OR #2 OR #3 OR #4 OR #5	2353
#9	#6 OR #7	2621
#10	#8 AND #9	10
